# A One-Year Radiographic Healing Assessment after Endodontic Microsurgery Using Cone-Beam Computed Tomographic Scans

**DOI:** 10.3390/jcm9113714

**Published:** 2020-11-19

**Authors:** Sumi Kang, Se-Won Ha, Ukseong Kim, Sunil Kim, Euiseong Kim

**Affiliations:** Microscope Center, Department of Conservative Dentistry and Oral Science Research Center, College of Dentistry, Yonsei University, Seoul 03722, Korea; smjty@yuhs.ac (S.K.); swha@yuhs.ac (S.-W.H.); ukpower@naver.com (U.K.); SEONE1@yuhs.ac (S.K.)

**Keywords:** apical surgery, radiographic healing, cone-bean computed tomography (CBCT), one-year follow up

## Abstract

This study aimed to evaluate one-year radiographic healing after endodontic microsurgery using CBCT with modified PENN 3D criteria and to compare the outcome with results evaluated using Molven’s criteria. A total of 107 teeth from 96 patients were evaluated one year after endodontic microsurgery by using CBCT scans with modified PENN 3D criteria and periapical radiographs with Molven’s criteria. Both preoperative and postoperative lesion volumes were calculated using ITK-SNAP (free software). Radiographic healing assessment using periapical radiographs and CBCT images, and preoperative and postoperative lesion volume measurements were performed independently by two examiners. The assessment using Molven’s criteria resulted in 75 complete healings, 18 incomplete healings, eight uncertain healings, and six unsatisfactory healings. Based on modified PENN 3D criteria, 64 teeth were categorized as complete healing, 29 teeth as limited healing, six teeth as uncertain healing, and eight teeth as unsatisfactory healing. With the one-year follow-up, CBCT scans showed a lower healing tendency than did periapical radiography. The volumes of apical radiolucency after the surgery were reduced by 77.7% on average at one-year follow up.

## 1. Introduction

The radiographic healing of endodontic microsurgery has been traditionally evaluated using the criteria defined by Rud et al. and Molven et al. [1,2]. Since Rud’s and Molven’s criteria were based on periapical radiographs, they also have some innate limitations of periapical radiographs in terms of their interpretation, such as background noise, distortion, and superimposition of anatomic structures. Despite these limitations, the criteria are still dominantly used for outcome assessment of apical surgery, especially for long-term follow-ups [3,4].

Cone-beam Computed Tomography (CBCT) has been widely used in endodontics [5,6]. In apical surgery, CBCT imaging is an important tool for treatment planning [7]. Although CBCT imaging has gained a wide reputation for diagnostics and treatment planning, it does not yet have the same impact on the assessment of endodontic treatment outcome because of economic aspects and concerns about radiation dose [8].

Based on the guidelines on the use of CBCT in Endodontics by European Society of Endodontology (ESE), CBCT is also considered with pre-surgical assessment prior to complex periradicular surgery [9]. In addition, several recent studies have suggested that CBCT has an impact on surgical outcome assessment. A study proposed new CBCT criteria based on an animal study correlated with CBCT and histology results and showed excellent repeatability and reproducibility on the criteria [10]. Another study assessed surgical healing using CBCT imaging with modified PENN 3D criteria during a mean follow-up period of 23.7 months [11]. The study also analyzed periapical lesion volumes before and after the surgery.

A previous study comparing the one-year follow-up and long-term follow-ups of endodontic microsurgery clinical outcomes using Rud’s criteria found no significant difference between the follow-up periods [3]. In this aspect, it is meaningful to evaluate radiographic healing using CBCT at one-year follow-up. Therefore, this study aimed to evaluate one-year radiographic healing after endodontic microsurgery using CBCT with modified PENN 3D criteria [12] and compare the outcomes with those evaluated using Molven’s criteria.

## 2. Materials and Methods

### 2.1. Case Selection

This study was approved by the Yonsei University Committee for Research on Human Subjects (No.2-2019-0076) and carried out in the Microscope Center of the Department of Conservative Dentistry, Yonsei University College of Dentistry and Dental Hospital, Seoul, South Korea. 

A clinical database was searched for patients who had received endodontic microsurgery between March 2011 and December 2018 performed by the same surgeon (E.K.). All surgeries were performed in an operating room using an operating microscope (OPMI pico; Carl Zeiss. Göttingen, Germany), as described in a previous study [13]. The root-end cavity was filled with ProRoot MTA (Dentsply, Tulsa, OK, USA), ENDOCEM MTA (Maruchi, Wonju, Korea), and RetroMTA (BioMTA, Seoul, Korea). Cases were selected using inclusion and exclusion criteria described as below.

The inclusion criteria were as follows

1. Endodontic microsurgery cases with both preoperative and 1 year follow-up CBCT images.
2. Teeth with surgical classification A-C defined by Kim and Kratchman [14].
3. Cases with both preoperative and one-year follow-up periapical radiographs.
4. Patient who had an intact restoration at follow-up.

The exclusion criteria were as follows:

1. Teeth with lesions connected to adjacent teeth.
2. Teeth with root fractures or perforations.
3. Teeth with lesion communicating with the alveolar crest and classes D-F defined by Kim and Kratchman [14].
4. Cases with the use of bone grafting or barrier materials.

### 2.2. Radiography

The periapical radiography was performed with a paralleling technique using an X-ray film holder (Rinn XCP; Dentsply, Elgin, IL, USA). The X-ray machine (CS2200; Carestream dental, Atlanta, GA, USA) was set at 60 kV and 7 mA, and exposure time for the periapical radiography ranged from 0.08 to 0.125 s (0.08 s for adult incisors and canines; 0.1 s for adult premolars; 0.125 s for adult molars). CBCT images were obtained using Alphard 3030 (Asahi Roentgen Ind Ltd., Kyoto, Japan) with a 0.1 mm^2^ voxel size and a limited field of view (51 × 51 mm^2^) with 17 s exposure time at 80 kV and 8 mA. 

### 2.3. Assessment of Healing Outcome (Healing Evaluation after Endodontic Microsurgery)

#### 2.3.1. 2D Radiographic Healing Assessment

In this study, 2D radiographic healing assessment referred to radiographic assessment using periapical radiographs and Molven’s criteria were used for the 2D assessment [1,2]. Periapical radiographic healing was evaluated independently by two examiners (S.K. and E.K.) with categories as follows: complete healing, incomplete healing, uncertain healing, and unsatisfactory healing. Both evaluators were calibrated and standardized before evaluation. Any disagreement cases were resolved by discussion until an agreement was reached. Complete healing and incomplete healing were classified as a success, and uncertain healing and unsatisfactory healing were classified as a failure based on Molven’s criteria. 

#### 2.3.2. 3D Radiographic Healing Assessment

In this study, 3D radiographic healing assessment was based on radiographic assessment using CBCT scans and Modified Penn 3D criteria were used for the 3D healing [11,12]. Healing assessment using CBCT images was performed independently by two examiners (S.H. and S.K.), and the two examiners discussed and reached an agreement in any disagreement cases. 

#### 2.3.3. Calculation of Lesion Volume in CBCT

**Figure 1:**
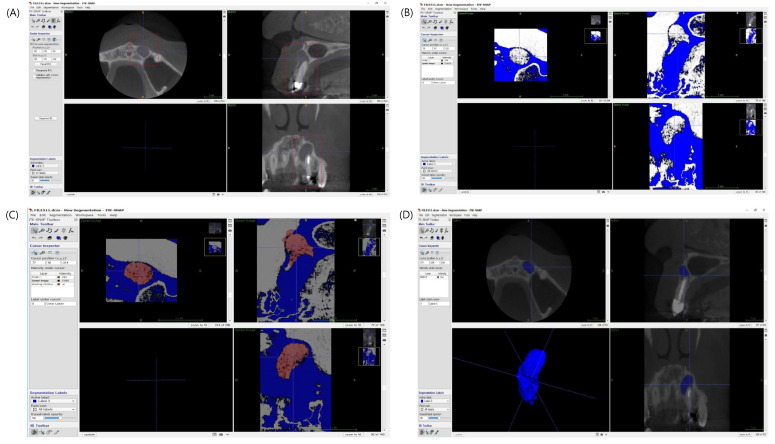
Lesion volume calculation using ITK-SNAP. (**A**) manual segmentation of a periapical defect in sagittal, axial and coronal views. (**B**) Selection of periapical defects using a grayscale value range selection tool. (**C**) Semiautomatic defect volume recognition and (**D**) reconstruction of 3D image on a lesion and calculation of the lesion volume.

For using modified PENN 3D criteria, the measurements of preoperative and postoperative volume in CBCT images were required for evaluating unsatisfactory healing, which referred to an enlarged or unchanged volume of the low-density area on postoperative CBCT scans. The lesion volumes preoperatively and at follow-up were calculated using ITK-SNAP (free software under the GNU General Public License developed by the National Institutes of Health, the US National Institute of Biomedical Imaging and BioEngineering, the US National Library of Medicine, the Universities of Pennsylvania and North Carolina, and an independent developer group), as described in a study [11] and summarized in Figure 1. Defect area segmentation and volume calculation were manipulated using the volumes at the highest resolution (slice thickness and intervals = 0.500 mm) in a Digital Imaging and Communication in Medicine (DICOM) exported from INFINITT PACS (a web-based 3D-enabled DICOM viewing station, INFINITT North America, Phillipsburg, NJ, USA).

### 2.4. Statistical Analysis

The 2D and 3D healing categories were assigned to scores, as shown in Table 1.

The Wilcoxon signed-rank test was applied to compare 2D and 3D radiographic healing assessment of the surgery outcome. Cohen kappa statistics was used for assessments of the agreement for postoperative 2D radiographic (S.K. and E.K.) and 3D radiographic (S.H. and S.K.) healing [15]. Inter-examiner agreements of preoperative and postoperative CBCT volume measurements were evaluated by intraclass correlation coefficient (ICC) [16]. All statistical analyses were performed using SPSS v.25 (IBM, Armonk, NY, USA) with a significance level of 0.05.

## 3. Results

Ninety-six patients and 107 teeth were included for this study; 11 patients underwent endodontic microsurgery twice on different teeth. Of the 96 patients, 31 were male and 65 were female with a mean age of 40.7 years. Of the 107 teeth, 84 teeth were in the maxilla and 23 were in the mandible, including 57 anterior, 28 premolars, 22 molars. Each root-end cavity of all treated teeth was filled with calcium silicate cement (ProRoot MTA, ENDOCEM MTA, and RetroMTA), most of which were RetroMTA (97, 90.7%). The distribution of the patients and the treated teeth for the final assessment was summarized in Table 2.

### 3.1. 2D Radiographic Healing Assessment

The results of the 2D healing assessment based on Molven’s criteria are summarized in Table 3.

Of 107 teeth, 75 teeth were classified as complete healing, 18 as incomplete healing, eight as uncertain healing and six as unsatisfactory healing. The success rate of the 2D healing assessment was 86.9% (93/107). On the postoperative periapical radiographic evaluations, the kappa value was 0.75, which showed a substantial agreement (S.K. and E.K.).

### 3.2. 3D Radiographic Healing Assessment

The results from radiographic evaluations using CBCT images are shown in Table 3. Sixty-four teeth were categorized as complete healing, 29 with limited healing, six with uncertain healing, and eight with unsatisfactory healing. The kappa value for evaluations of one-year postoperative CBCT scans was 0.93, which indicated an almost perfect agreement.

The ICC was 0.953 (95% confidence interval, 0.932–0.968) for the preoperative volume measurements and 0.928 (95% confidence interval, 0.893–0.950) for the postoperative volume measurements, which were significantly different with a level of significance of 0.05. The ICC values of both preoperative and postoperative volume measurements showed excellent agreement between the two examiners (S.H. and W.K.). The mean preoperative lesion volume was 136.6 mm^3^ (*n* = 107, SD ± 139.5 mm^3^) and the mean postoperative lesion volume was 30.5 mm^3^ at the one-year follow up (*n* = 107, SD ± 65.6 mm^3^). The lesion volume had reduced, on average, by 77.7% during a one-year follow-up.

### 3.3. Comparison of 2D and 3D Healing

As shown in Table 1, a scoring system was used for 2D and 3D healing assessment of the apical surgery: score 1 for complete healing in 2D and 3D, score 2 for incomplete healing in 2D and limited healing in 3D, score 3 for uncertain healing in 2D and 3D, and score 4 for unsatisfactory healing in 2D and 3D. The mean score was 1.49 for 2D radiographic healing and 1.61 for 3D radiographic healing, respectively. The mean score between 2D and 3D radiographic healing was compared using Wilcoxon signed-rank test, and the difference was statistically significant (*z* = −3.6, *p* < 0.05).

**Figure 2:**
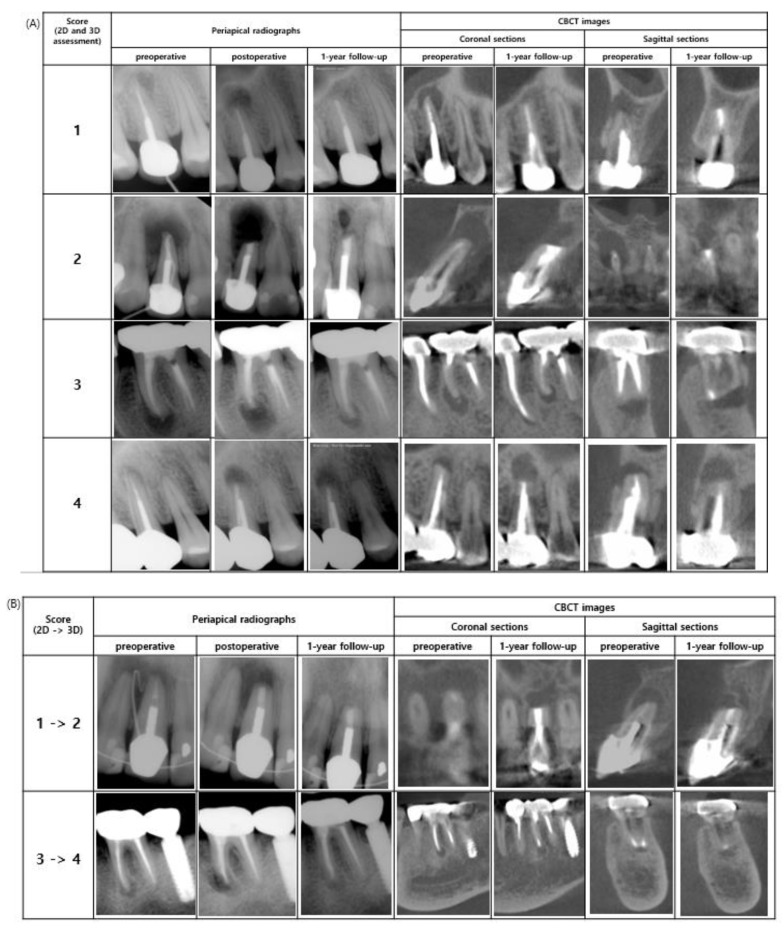
(**A**) Examples of agreement cases. The images in each row were scored 1, 2, 3 and 4 resulted from both 2D and 3D assessments. (**B**) Examples of disagreement cases. The images at the first row were scored 1 from 2D assessment, but scored 2 from 3D assessment. The images at the second row were scored 3 from 2D assessment, but 4 from 3D assessment.

For the analysis of 2D and 3D radiographic evaluation, scores from 2D and 3D radiographic healing assessment were compared, as shown in Table 4 and Table 5. When the case had the same score resulting from the 2D and 3D radiographic healing assessment, the case was considered as an agreement case; otherwise, it was considered as a disagreement case. There were 94 agreement cases and 13 disagreement cases, and the agreement rate was 87.6% (94/107) (Table 4). The details of agreement and disagreement cases are summarized in Table 5. Of the 13 disagreement cases, 11 cases were scored as 1 in 2D assessment but scored as 2 in 3D assessment (84.6%, 11/13); two cases were scored as 3 in 2D assessment but scored as 4 in 3D assessment. Additionally, examples of agreement and disagreement cases are shown in Figure 2A,B, respectively.

## 4. Discussion

Molven’s and Rud’s criteria have been predominant methods for evaluating periapical radiographic healing outcomes of endodontic microsurgery. However, the evaluation using periapical radiographs was limited to the mesiodistal plane of the tooth, which could provide little information on periapical lesion. With CBCT, periapical lesions and teeth can be evaluated buccolingually as well as mesiodistally, which could provide more data on the tooth and the lesion. In this regard, preoperative CBCT is required to estimate and predict the process before endodontic surgery. A study found that using Rud’s criteria at the one-year follow-up may be acceptable to predict the long-term prognosis [3]. Thus, it is necessary to evaluate radiographic healing using CBCT at a one-year follow up. In this study, modified PENN 3D criteria was employed for evaluating 3D radiographic healing because the method of rating process on the healing of the treated teeth was similar to Molven’s and Rud’s criteria (Table 1).

With radiation concern, the patients in this study underwent CBCT in the limited field of view mode with an effective dose of 81.46 ± 0.13 μSv [17], which was 30% of the effective does at a standard setting, whose effective dose was 273.7 ± 0.87 μSv [17]. Although the mode of CBCT was set at a limited value, the irradiation issue still remained because the effective dose was higher than that of panoramic radiography, which was 6.39 ± 0.01 μSv [17]. All patients who were planning to undergo the apical surgery were easily accepted to take CBCT preoperatively, but most of them disagreed with undergoing CBCT at follow-ups. Thus, from 2011 to 2018, 107 teeth of 96 patients were collected. Regarding the period, the number of the study population was not representative of the overall population, but it is sufficient when considering the population of the previous studies on healing assessment using CBCT [11,18].

The 2D and 3D radiographic healing assessment were performed independently with two examiners and showed a substantial agreement for 2D assessment and an almost perfect agreement for 3D assessment between the examiners. When the results of 2D and 3D radiographic healing assessment were divided into success and failure according to Molven’s criteria, the success rate was 86.9% (93/107) for both assessments. However, the distribution of 2D and 3D radiographic healing assessment was significantly different, as shown in Table 3. The total score of the 2D radiographic healing assessment was 159, and that of the 3D assessment was 172, which showed a worse outcome tendency of 3D evaluation than 2D evaluation. This result was consistent with some studies, which also found that evaluation using CBCT resulted in a worse outcome than evaluation using periapical radiography [11,18].

A previous study found that the volume of the preoperative periapical lesion had a significant effect on the outcome of endodontic microsurgery [19]. Volume measurement of periapical lesions is one of the advantages of evaluating radiographic lesion healing process using CBCT. The method of volume measurement described by Schloss et al. enables measurement of periapical lesion volume as a whole with a couple of computer software [11]. For more precise comparison of changes in periapical lesion volume before and after the surgery, immediate postoperative CBCT would be better taken because buccal bone was removed for access of the root and root tips were resected during the surgery, which could have led to differences in volume measurement and healing process. Therefore, removing buccal bone and root tips could not be counted when evaluating the volume measurement and healing process. Since immediate postoperative CBCTs have not been reasonably accepted so far because of the radiation concern, this study evaluated volume changes before and after the apical surgery using preoperative CBCT and one-year follow-up CBCT. Both preoperative and follow-up volumes were calculated independently by two examiners independently, whose ICC showed an excellent agreement. The average volume measurements were 136.6 mm^3^ before apical surgery and 30.5 mm^3^ at one-year follow-up, which had decreased by 77.7%.

The individual scores resulting from 2D and 3D radiographic healing assessments were compared and categorized as agreement cases and disagreement cases, as summarized in Table 4 and Table 5. There were 94 agreement cases, and the agreement rate was 87.6% (94/107). A previous study reported that mean overall agreement was 59.5% when comparing 2D and 3D radiography [18], which had different criteria and methods for 3D radiographic healing assessment.

To analyze the differences between 2D and 3D assessments, disagreement cases were additionally investigated. Of the 13 disagreement cases, the majority of them (11 cases) was scored as 1 in 2D assessment but scored as 2 in 3D assessment. They fell into 2C or 2D of 3D radiographic healing assessment criteria, even though they showed reformation of periodontal space around the resected root surfaces. This discrepancy resulted from the fact that CBCT scans can be used to assess additional buccolingual sections, where the cortical bone plate can be evaluated. Cortical bone healing could not be evaluated by periapical radiography, which could lead to the difference between the two assessments. A long-term study of radiographic healing after apical surgery using CBCT found that new hard tissue formation at the resection plane and within the former apical defect was advanced at five years, and the reestablishment of the cortical bone plate lagged behind [20]. This finding could be the one reason for the disagreement cases, which were assessed as ‘complete healing’ in a periapical radiograph but were assessed as “limited healing” in CBCT images.

The remaining two cases with a score of 3 in the 2D assessment but 4 in the 3D assessment, were caused by the volumetric analysis. Unchanged or enlarged the volume of the low-density area was evaluated as “unsatisfactory healing” when evaluated with modified PENN 3D criteria. Given the excellent inter-examiner agreement in the volume measurement, cases with an increased volume of lesion at the follow-up were categorized as “unsatisfactory healing” in this study. Meanwhile, the “unsatisfactory healing” was more quantitatively categorized than with Molven’s “unsatisfactory healing” by using the volume measurement, because angulation change could be occurred when taking periapical radiography preoperatively and at follow-ups, and this could lead to deviation in rarefaction area. However, limitation remains on calculating volumetric changes when comparing volumes preoperatively and at one-year follow-up, not comparing volumes immediately after apical surgery and at one-year follow-up. As shown in Figure 2B, the mesial root of the left mandibular first molar was evaluated as “uncertain healing” with Molven’s criteria but assessed as “unsatisfactory healing” with modified PENN 3D criteria because the postoperative volumes were increased compared with preoperative volumes (from 22.1 mm^3^ to 48.0 mm^3^). The tooth could be evaluated as “limited healing” in 3D assessment, except for the volume changes. Given that volumetric analysis is one of the advantages on apical surgery outcome evaluation using CBCT images, evaluation of volume changes should be considered as a comparison between post-operation (immediately after apical surgery) and at follow-up.

## 5. Conclusions

The distributions resulting from radiographic healing assessments of endodontic microsurgery using modified PENN 3D criteria were significantly different from those using Molven’s criteria. With the one-year follow-up, CBCT scans showed a lower healing tendency than did periapical radiography. The volumes of apical radiolucency after the surgery were reduced by 77.7% on average at one-year follow up.

## Figures and Tables

**Table 1:** A scoring system for 2D and 3D radiographic healing assessment of the endodontic microsurgery.

Scores	2D Radiographic Healing Assessment Criteria ^†^	3D Radiographic Healing Assessment Criteria ^‡^
1	Complete healing	Complete healing
	(A) Re-formation of periodontal space of normal width and lamina dura to be followed around the apex. (B) Slight increase in width of apical periodontal space, but less than twice the width of non-involved parts of the root. (C) Tiny defect in the lamina dura adjacent to the root filling. (D) Complete bone repair; bone bordering the apical area does not have the same density as surrounding non-involved bone. (E) Complete bone repair; no apical periodontal space can be discerned.	(A) Re-formation of periodontal space of normal width and lamina dura over the entire resected and un-resected root surfaces. (B) Slight increase in width of apical periodontal space over the resected root surface, but less than twice the width of non-involved parts of the root. (C) Small defect in the lamina dura surrounding the root-end filling. (D) Complete bone repair with discernible lamina dura; bone bordering the apical area does not have the same density as surrounding non-involved bone. (E) Complete bone repair. Hard tissue covering the resected root-end surface completely. No apical periodontal space can be discerned.
2	Incomplete healing (scar tissue)	Limited healing
	Rarefaction has decreased in size or remained stationary, and is characterized by one or more of the following findings: (A) Bone structure are recognized within the rarefaction. (B) The periphery of the rarefaction is irregular and may be demarcated by a compact bone border. (C) The rarefaction is located asymmetrically around the apex. (D) The connection of the rarefaction with the periodontal space is angular.	Complete healing can be observed in immediate vicinity of the resected root surface, but the site demonstrates one of the following conditions: (A) The continuity of the cortical plate is interrupted by an area of lower density. (B) A low density area remains asymmetrically located around the apex of has an angular connection with the periodontal space. (C) Bone has not fully formed in the area of the former access osteotomy. (D) In areas with pre-existing periodontal disease or physiologic fenestrations un-resected root surfaces do not demonstrate bone coverage and/or periodontal reattachment.
3	Uncertain healing	Uncertain healing
	The rarefaction has decreased in size, and with one or more of the following characteristics: (A) The radiolucency is larger than twice the width of the periodontal space. (B) The radiolucency is bordered by lamina-dura like bone structures. (C) The radiolucency has a circular or semicircular periphery. (D) The radiolucency is located symmetrically around the apex as a funnel-shaped extension of the periodontal spate. (E) Bony structures are discernible within the bony cavity.	The volume of the low density area appears decreased and demonstrates one of the following conditions: (A) The thickness is larger than twice the width of the periodontal space. (B) The location is symmetrically around the apex as a funnel-shaped extension of the periodontal space.
4	Unsatisfactory healing	Unsatisfactory healing
	The rarefaction has enlarged of is unchanged.	The volume of the low density area appears enlarged or unchanged.

^†^, ^‡^: The assessments were based on Molven’s criteria for 2D healing [1,2] and modified Penn criteria for 3D healing [11,12], respectively.

**Table 2:** Case distribution of patients (*n* = 96) and treated teeth (*n* = 107).

	Cases, *n* (%)
Patient total	96
Sex	
Male	31 (32.3)
Female	65 (67.7)
Age (y) *	
<20	2 (2.1)
21–30	26 (27.1)
31–40	22 (22.9)
41–50	17 (17.7)
51–60	15 (15.6)
>60	14 (14.6)
Treated teeth total	107
Tooth position	
Maxillary anterior	50 (46.7)
Maxillary premolar	25 (23.4)
Maxillary molar	9 (8.4)
Mandibular anterior	7 (6.5)
Mandibular premolar	3 (2.5)
Mandibular molar	13 (12.1)
Retrograde filling materials	
ProRoot MTA	1 (0.9)
ENDOCEM MTA	9 (8.4)
RetroMTA	97 (90.7)

* Age at the time of apical surgery.

**Table 3:** A summary of results from 2D and 3D radiographic healing assessment of one-year after the endodontic microsurgery.

	2D *n* (%)	3D *n* (%)
1 (complete healing)	75 (70.1)	64 (59.8)
2 (incomplete (limited) healing)	18 (16.8)	29 (27.1)
3 (uncertain healing)	8 (7.5)	6 (5.6)
4 (unsatisfactory healing)	6 (5.6)	8 (7.5)
Total	107	

**Table 4:** An analysis of agreement and disagreement between 2D and 3D radiographic healing assessment of one-year after the apical surgery. The total number of cases and rates of agreement and disagreement between 2D and 3D radiographic evaluations.

	*n* (%)
Agreement between 2D and 3D	94 (87.6)
Disagreement between 2D and 3D	13 (12.4)

**Table 5:** The details on agreement and disagreement between 2D and 3D radiographic evaluations. For example, ‘1→2′ indicates that it was scored as 1 in 2D assessment, but scored as 2 in 3D assessment, and this case was considered as a disagreement case.

2D→3D	*n*
1→1	64
1→2	11
1→3	0
1→4	0
2→1	0
2→2	18
2→3	0
2→4	0
3→1	0
3→2	0
3→3	6
3→4	2
4→1	0
4→2	0
4→3	0
4→4	6
